# Logatome Discrimination in Cochlear Implant Users: Subjective Tests Compared to the Mismatch Negativity

**DOI:** 10.1100/tsw.2010.28

**Published:** 2010-02-19

**Authors:** Torsten Rahne, Michael Ziese, Dorothea Rostalski, Roland Mühler

**Affiliations:** ^1^ Department of Otolaryngology, Martin-Luther-University Halle-Wittenberg, Germany; ^2^ Department of Experimental Audiology and Medical Physics, Otto-von-Guericke University, Magdeburg, Germany; ^3^ Department of Otolaryngology, Otto-von-Guericke University, Magdeburg, Germany

**Keywords:** mismatch negativity (MMN), cochlear implant (CI), logatome discrimination, speech tests

## Abstract

This paper describes a logatome discrimination test for the assessment of speech perception in cochlear implant users (CI users), based on a multilingual speech database, the Oldenburg Logatome Corpus, which was originally recorded for the comparison of human and automated speech recognition. The logatome discrimination task is based on the presentation of 100 logatome pairs (i.e., nonsense syllables) with balanced representations of alternating “vowel-replacement” and “consonant-replacement” paradigms in order to assess phoneme confusions. Thirteen adult normal hearing listeners and eight adult CI users, including both good and poor performers, were included in the study and completed the test after their speech intelligibility abilities were evaluated with an established sentence test in noise. Furthermore, the discrimination abilities were measured electrophysiologically by recording the mismatch negativity (MMN) as a component of auditory event-related potentials. The results show a clear MMN response only for normal hearing listeners and CI users with good performance, correlating with their logatome discrimination abilities. Higher discrimination scores for vowel-replacement paradigms than for the consonant-replacement paradigms were found. We conclude that the logatome discrimination test is well suited to monitor the speech perception skills of CI users. Due to the large number of available spoken logatome items, the Oldenburg Logatome Corpus appears to provide a useful and powerful basis for further development of speech perception tests for CI users.

## INTRODUCTION

For several decades, adults and children who suffer from severe-to-profound sensorineural hearing loss have been able to benefit from a cochlear implant (CI). Electrical stimulation of the auditory nerve partly restores hearing ability. However, speech perception skills postimplant can have high variability across individuals[1,2,3].

Postimplant, speech intelligibility measures have predominantly relied on behavioral tests. These tests were developed in isolation of the respective language areas, mostly referring to genuine words and sentences. Representative examples are the Freiburger monosyllabic word test (FMW) for German-speaking subjects[4] and the Hearing in Noise Test (HINT) for English-speaking subjects[5].

Such speech intelligibility tests are very limited for the assessment of CI users with prelingual deafness or prolonged periods of deafness, as they generally do not have good perception abilities for naturally spoken language. In addition, children CI users, particularly the very young, in many cases are not able to perform behavioral speech intelligibility tests because of their cognitive developmental status. Thus, objective measures are needed that do not demand the subjects' attention and cooperation, or a certain level of speech and language skills.

Electrophysiological methods have been proposed as an alternative and supplementary objective measure to behavioral data. Observations to date show evidence of correlations between speech intelligibility ability and objective measures for auditory-evoked event-related potentials, N1, P2, P3[6], the acoustic change complex[7,8], and the mismatch negativity (MMN) component[9,10]. Since other studies could not show such correlation, the current study was conducted to provide a compelling reason for using electrophysiological measures[11].

The MMN component of event-related brain potentials (ERPs), which provides a noninvasive electrophysiological measure of cortical auditory processing, reflects the outcome of a change detection process that is based on the memory of sound regularities (often called the "standard") in ongoing auditory input[12,13]. Incoming sounds that deviate from the neural representation of the standard sound in simple auditory features, such as frequency, intensity, tone duration, or spatial location, as well as changes in more complex features such as syllables, elicit a MMN[9,14,15], for review, see[13,16]. MMN is typically observed with a peak latency of 150 msec from the time that the deviation is detected. Thus, MMN represents an early process of deviance detection based on a memory of the previous sound stimulation. Its elicitation does not require participants to detect the deviant sounds actively[17,18,19,20]. The MMN thus seems to be an appropriate tool to investigate preattentive discrimination in uncooperative patients, e.g., young children or CI listeners who are unable to describe what they are hearing[9,21,22]. As found in Ponton and Don[23] and Kraus et al.[14], the measurement of MMN responses also holds promise as a useful objective measure for the evaluation of CI function for the broader group of CI users and for further research of neurophysiological central processes underlying speech perception. Research by Groenen et al.[6,24] shows evidence of correlation between speech perception abilities and the MMN responses. Investigating late and cognitive-evoked potentials in children CI users, a correlation between the amplitudes and the sentence recognition scores was also observed[25,26]. To apply the MMN as a tool to investigate the discrimination abilities of prelingual deafened patients, it is important to state that ERPs are obtained in prelingual CI users as well[27]. In CI users, a growing MMN amplitude was observed during the course of CI use[28]. Beside this training effect, the latency shift of the MMN with increasing age has to be considered[10]. To date, the sensitivity and specificity of the MMN for the ability to discriminate speech sounds has not been verified[29]. Nevertheless, the MMN component may function as a complementary clinical tool to assess auditory sensitivity objectively, although further research is required[8,30,31,32].

On the other hand, there is also a need for behavioral tests designed to measure more basic speech perception skills, such as discrimination of speech pattern contrast[33], speakers, or logatomes. In general, logatomes are nonsense syllables, e.g., used for analyzing the confusion of phonemes by hearing-impaired listeners[34].

In this paper, we describe a new logatome discrimination test. Thereby, we benefit from use of the Oldenburg Logatome (OLLO) Corpus, which was described recently by Wesker et al.[35], and was originally recorded for the comparison of human and automated speech recognition. The logatomes of the OLLO Corpus consist of either consonant–vowel–consonant (CVC) or vowel–consonant–vowel (VCV) paradigms. All logatomes were pronounced with the speaker-independent variables of speaking rate (fast, normal, slow), speaker effort (low, normal, high), and speaker style (statement, question), resulting in a total of 2,700 logatome items per speaker. All recordings were normalized to 99% amplitude. Thus, the different speaker effort varies, mainly the frequency spectrum of the logatomes. The German element of the Corpus covers four different regional dialects (no dialect, Bavarian, East Frisian, and Eastphalian), with 10 speakers (five male, five female) per dialect. Speech data were recorded in sound-isolated rooms at three different sites in Germany (Oldenburg, Magdeburg, and Munich). For a more detailed description, see[35].

Our concept of testing low-level speech perception abilities is based on the usage of subsets of the OLLO Corpus. This includes three improvements over traditional speech intelligibility tests. First, the test material consists of speech material that is natural, i.e., recorded from nonprofessional speakers of different dialect regions; hence, approaching real hearing situations more closely than test materials recorded by only one professional speaker. Second, the stimuli are logatomes that are considered appropriate for speech audiometry measures: based on the established significant correlation between logatome perception and pure-tone audiometric thresholds at 1, 2, 3, and 4 kHz[34]. In view of recent work, using the logatomes of the OLLO Corpus to develop a statistically powerful, speaker discrimination test for CI users[36], the items were considered suitable for development of a logatome discrimination task also. The third improvement is related to the advantage of measuring speech discrimination ability instead of speech intelligibility, which does not require a certain level of speech and language skills to perform the task. As such, this is considered a very valuable addition to the clinical speech audiometric test battery to assess the development of hearing skills even in the case of prelingual deafened CI users and thus provide indispensable information for the ongoing clinical management.

Using this new logatome discrimination test, we measured the discrimination ability of a group of normal hearing adult subjects and a group of adult CI users, and compare their outcomes with their results on the commonly used Oldenburg Sentence Test (OLSA), measuring speech intelligibility in competing background noise. The OLSA was designed and evaluated for the German-speaking language area[37,38]. Electrophysiological examination of MMN responses elicited by logatome speech stimuli was also performed. Subsequently, MMN responses and the results for the newly developed logatome discrimination test were compared and examined for correlation.

## METHODS

A prospective comparative study was performed in two subgroups of subjects in order to assess and compare performance on conventional behavioral speech tests, with a newly developed speech discrimination task and objective measures to assess auditory hearing skills.

Evaluation measures included the OLSA, electrophysiological measurement of MMN responses, and logatome discrimination test.

### Subjects

Thirteen adults (seven females, six males; 19–35 years of age) with normal hearing participated in the study. Subjects were enrolled following audiometric hearing threshold screening to confirm hearing thresholds for pure tones <10 dB HL (hearing level) for the octave frequencies 125–8,000 Hz inclusively.

Eight adults (six females, two males; 48–71 years of age) using a unilateral Nucleus® CI (models CI22M, CI24M, or CI24RE); fitted with a SPRINT, 3G, SPECTRA, or Freedom sound processor; with at least 1-year experience with their current processor were enrolled in the study. Subject demographics are shown in Table 1. All subjects provided written informed consent for their participation in the study. The study was approved by the Ethics Committee of the University of Magdeburg and performed in accordance with the 2004 Declaration of Helsinki for the conduct of medical research on patients.

**TABLE 1**
**CI User Demographics**

| Subject No. | Sex | Ear implanted | Age at implant (years) | Device | Speech processor | Strategy | Deafness onset | Duration of CI use (years) |
|---|---|---|---|---|---|---|---|---|
| 1 | F | R | 70 | CI24RE | Freedom | ACE | postlingual | 1.5 |
| 2 | F | R | 56 | CI24RE | Freedom | ACE | postlingual | 1.5 |
| 3 | F | R | 50 | CI22M | 3G | ACE | postlingual | 15 |
| 4 | M | R | 33 | CI22M | Spectra | SPEAK | perilingual | 14 |
| 5 | F | R | 53 | CI22M | Spectra | SPEAK | postlingual | 14 |
| 6 | M | R | 63 | CI24RE | Freedom | ACE | postlingual | 0.5 |
| 7 | F | R | 47 | CI24M | Sprint | SPEAK | perilingual | 11 |
| 8 | F | R | 53 | CI24RE | Freedom | ACE | postlingual | 1.5 |

## Materials

### *OLSA*

The subjects were seated in a sound-attenuated room. Stimuli were presented in free field, originating from a PC with a studio-quality sound card (RME-HDSP-9632), a studio-quality power amplifier (MAM-PA200), and a single loudspeaker (Tannoy Reveal), positioned 1.5 m in front of the subject. A constant noise level of 65 dB SPL (sound pressure level) was used with an adaptive level for the speech stimuli commencing with 75 dB SPL and altered depending on the individual’s response.

One test list, consisting of 30 sentences, was randomly selected via the software for presentation. All sentences consisting of a subject–verb–numeral–adjective–object five-word structure are automatically created from a 50-word inventory. As such, sentences cannot be memorized or anticipated by the subject due to contextual factors. Sentences were superimposed by speech-simulating noise and presented successively as a closed-set test, i.e., the subject’s task was to select the correct sentence (every word correct) out of 10 possible responses for every word. The response was given by clicking on the choices displayed on a touch-screen display. Using an adaptive speech level algorithm, the speech reception threshold for a 50% correct word score was determined to identify speech intelligibility for each individual, and reported as the corresponding signal-to-noise ratio (dB SNR).

### *Electrophysiological Experiment*

Three logatomes (/gag/, /bab/, and /geg/) of the OLLO Corpus were used for electrophysiological measurements. They were spoken by one German speaker with no dialect, normal speaking effort, speaking rate, and statement speaking style.

The stimuli were presented in two oddball conditions. In the *vowel-replacement* condition, the series of “standard” stimuli (/gag/) was randomly replaced by “deviant” stimuli (/geg/). In the *consonant-replacement* condition, the logatome /bab/ was used as “deviant” to the “standard” /gag/.

Totally, in each condition, 220 deviants were presented with a probability of 15%, whereas the “deviant” stimuli were separated by at least four “standard” stimuli. Additionally, two control conditions containing 450 of the /bab/ or /geg/ stimuli, respectively, were run to test if the MMN is absent in response to that same stimulus when presented alone[39]. The stimulus onset asynchrony was 1 sec; the total stimulation time of 45 min was separated in six blocks with intermediate breaks.

The experiment was performed on a PC using the PRESENTATION software (Neurobehavioral Systems, Albany, NY) and insert earphones (E-A-RTONE 3A) for normal hearing subjects or free-field presentation for CI users for the best possible adjustment of the SPL. The stimuli were calibrated using a programmable attenuator (gPAH, g.tech medical Engineering, Graz, Austria) at 75 dB SPL and presented in the free field in a sound-attenuated booth, originating from a PC with a sound card and loudspeaker (Hi-TEX), positioned 1.5 m in front of the subject.

EEG was continuously recorded with a Neuroscan Synamps AC-coupled amplifier (0.05–200 Hz bandwidth; sampling rate: 1 kHz) using electrodes placed at Fz according to the International 10–20 System, plus the left and right mastoids, and referred to the nose. Impedances were kept below 5 kOhms.

The EEG at Fz and the mastoid channels were digitally filtered (1–15 Hz bandpass, 24 dB/oct. roll-off) and then epoched relative to reference marker positions according to the stimulus type (standard, deviant, and controls). Epochs were 400 msec, starting from 100 msec before and ending 300 msec after the onset of the stimulus. An artifact rejection criterion was set at ±50 µV and applied after the epochs were baseline corrected on a prestimulus range of 100 msec. The "deviant" and "control" ERPs were calculated as average of the relative epochs. Difference waveforms were obtained by subtracting the ERPs elicited by the "deviant" stimuli from the ERPs elicited by the "control" stimuli. For every subject and both deviant types, the MMN response was identified as minimum amplitude in the latency range of 100–250 msec of the difference waveforms. The amplitudes of the "deviant" and "control" averages were also determined at this latency time point. A paired *t*-test was performed to determine the significance of the difference between "deviant" and "control" responses.

### *Logatome Discrimination Test*

For auditory stimulation, a subset of the OLLO Corpus was used. Recording conditions, speaker instructions, and postprocessing of the recorded material are described in detail in Wesker et al.[35]. The entire OLLO Corpus is approximately 4.6 GB and available publicly, including a detailed description, word lists, labeling files, technical specifications, and calibration data. It can be downloaded for free from http://sirius.physik.uni-oldenburg.de.

For the logatome discrimination test described here, we selected the 70 logatomes (L071–L150) of CVC structure, recorded by one male speaker (S03M) with a normal articulation characteristic (V2). The stimuli were calibrated using a programmable attenuator (gPAH, g.tech medical Engineering, Graz, Austria) at 75 dB SPL and presented in the free field in a sound-attenuated booth, originating from a PC with a sound card and loudspeaker (Hi-TEX), positioned 1.5 m in front of the subject. Through the use of a software program written in DELPHI, 100 logatome pairs (50 same, 50 different) were randomly selected and presented in same-different combinations. After the presentation of every logatome pair, the subject's perception was recorded by selection of a two-alternative forced-choice (2AFC), pressing button #1 for a "same" impression and button #2 for a "different" impression. Logatome pairs were presented without feedback regarding correct or incorrect responses. The stimuli and the subjects' responses were stored in a text file, and evaluated by calculating the "hits", "misses", "false alarms", and "correct rejections" to determine the sensitivity index *d'* for every subject.

## RESULTS

## OLSA

Fig. 1 shows the results of the OLSA. All participants with normal hearing could perform the OLSA, identifying 50% of the test material when the noise was louder than the target speech ($M$ = –6.00 dB SNR, $SD$ = 0.51 dB SNR). However, only four of the eight CI users were able to perform the OLSA in noise ($M$ = 4.53 dB SNR, $SD$ = 0.39 dB SNR). Compared to the normal hearing subjects, the required speech SNR to achieve their speech reception threshold ($M$ = 4.53 dB SNR, $SD$ = 0.39 dB SNR) was significantly

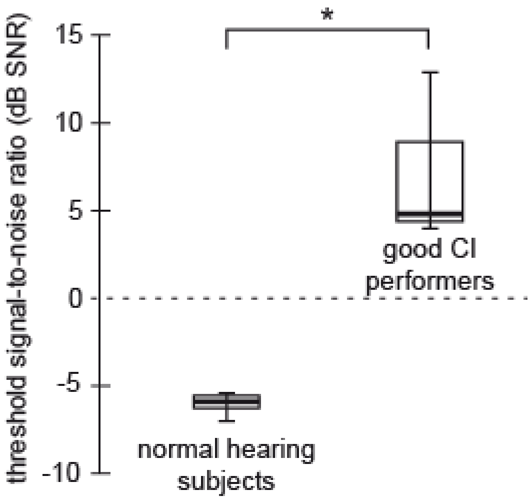

**FIGURE 1.** Threshold SNR as outcome of the OLSA, displayed as a boxplot for the normal hearing subjects and the good CI performers. The SNR is significantly poorer for the good CI performers (*$p < 0.01$). The poor CI performers were not able to perform the test.

higher ($t$ (13) = 10.45, $p < 0.01$). This group was labeled as “good CI performers”. For the remaining four CI users, labeled as “poor CI performers”, the tests were interrupted due to a lack of specificity approaching floor effects. There was no correlation observed between the age of the participants or their onset of deafness and the speech intelligibility results.

## Electrophysiological Experiment

Fig. 2a shows the group average ERPs elicited by the “deviant” and the “control” stimuli for the normal hearing subjects, and the good and poor CI performers. For normal hearing subjects and good CI performers, a clear P1-N1 waveform is elicited in response to the consonant-replacement logatome paradigm as well as in response to the vowel-replacement logatome paradigm. No clear P1-N1 waveform is seen for the poor CI performers. With the exception of the poor CI performers, a statistically significant difference is observed between the “deviant” and “control” responses ($t$-tests, $p < 0.05$), standing for a MMN response.

Fig. 2b shows the difference ERP waveforms for the consonant- and vowel-replacement logatome paradigms. After confirming the normal distribution and homogeneity of the ERP amplitudes (Mauchly’s test, $p > 0.05$), a two-way mixed ANOVA (discrimination task: two levels, consonant replacement and vowel replacement; speech intelligibility, three levels: normal hearing, good CI performers, and poor CI performers) was performed on the amplitude of the MMN peaks. There was a significant main effect of the speech intelligibility ($F$ (2, 32) = 14.97, $p < 0.001$). Posthoc tests revealed significantly larger MMN amplitudes for subjects with normal hearing compared to that observed for poor CI performers. In comparison, the good CI performers also revealed larger MMN amplitudes than observed for the poor CI performers (Bonferroni tests, all $p < 0.01$). Neither the main effect of discrimination task ($F$ (1, 32) = 0.68, *ns*), nor the discrimination task × speech intelligibility interaction ($F$ (2, 32) = 1.13, *ns*) were significant.

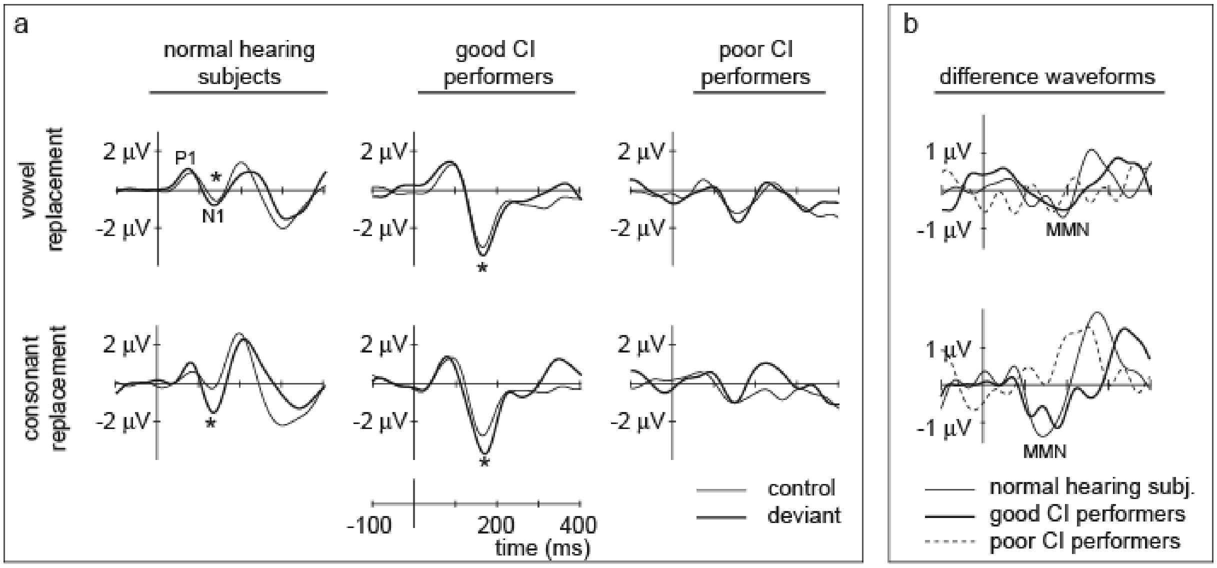

**FIGURE 2.** (a) Group average ERP waveforms for the normal hearing subjects, and the poor and good CI performers, recorded at the electrode site Fz. A negative difference between the "deviant" (bold line) and the "control" (thin line) waveform in a latency range between 100 and 250 msec was identified as MMN. For the normal hearing subjects and the good CI performers, a clear MMN was observed for both the vowel- and the consonant-discrimination task of CVC logatomes. No significant difference was found for the poor CI performers (*$p < 0.05$). (b) The group average difference waveforms show the different MMN potentials for the vowel- and consonant-replacement logatome paradigms.

## Logatome Discrimination Test

All except one CI user finished the logatome discrimination test. For normal hearing subjects, the overall hit rate was mostly 100%. For CI users, the hit rate varied from chance level (50%) to 100%. Fig. 3 shows the measured sensitivity index *d'* of the logatome discrimination test for the normal hearing group as well as for the good and poor CI performers' groups. Clear differences between the groups are discernible.

A two-way mixed ANOVA (discrimination task: two levels, consonant and vowel replacement; speech intelligibility, three levels: normal hearing, good CI performers, and poor CI performers) was performed on the sensitivity index *d'* of the logatome discrimination test after confirming the normal distribution and homogeneity of the sensitivity index (Mauchly's test, $p > 0.05$). The results show that the *d'* of both the two discrimination tasks ($F$ (1, 16) = 46.50, $p < 0.01$) and the three speech intelligibility groups differed significantly ($F$ (2, 16) = 95.51, $p < 0.01$). A significant interaction of the discrimination tasks and speech intelligibility was found ($F$ (2, 16) = 24.64, $p < 0.01$).

Posthoc tests revealed that, over all subjects, the discrimination ability was higher for the normal hearing subjects compared to that for the good and poor CI performers. The poor CI performers revealed the poorest discrimination abilities. The posthoc test of the interaction between the discrimination task and speech intelligibility revealed a significantly higher *d'* for the vowel replacement compared to the consonant replacement only for the good CI performers (Bonferroni test, all $p < 0.05$).

Fig. 3 also displays the individual MMN amplitudes compared to the results of the logatome discrimination test. While the sensitivity index was different between all subject groups, significantly smaller MMN amplitudes were only obtained for the poor CI performers group compared to the good CI performers and normal hearing subjects group.

## DISCUSSION

The results of the experiments presented in this paper demonstrate that the OLLO Corpus can be used to design an effective logatome discrimination test for CI users. With its studio-quality recordings of

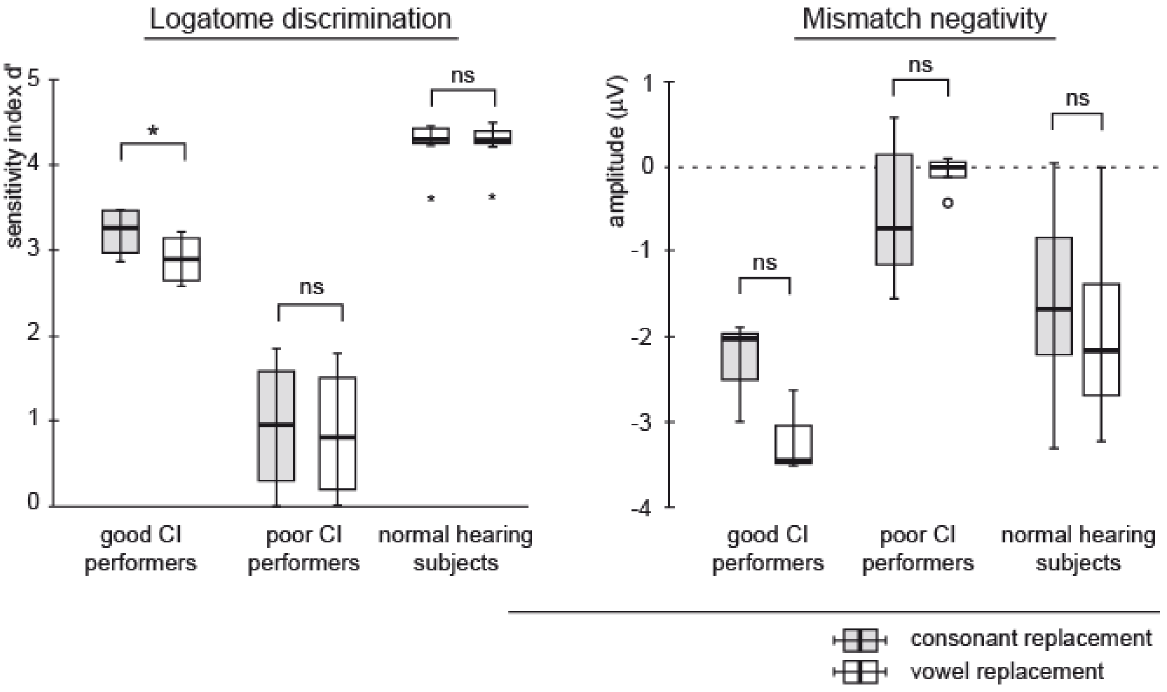

**FIGURE 3.** Sensitivity index *d'* of the logatome discrimination test (left) and individual MMN amplitudes (right), displayed as boxplots for the three speech intelligibility groups. Please note the clustering of the *d'* between the speech intelligibility groups. Differences between vowel- and consonant-replacement logatome paradigms are significant for the psychophysical measure (*d'*) in the good CI performers group (*$p < 0.01$) only.

logatomes from 40 German speakers, it is the first corpus that is available for the German-speaking region. Recently published work also reports the first speaker discrimination test drawing its stimuli from this corpus[36].

Only the good CI performers were able to perform the OLSA. As this is a speech intelligibility test, this demonstrates the clinical need for more basic speech perception tests as demonstrated by the newly developed logatome discrimination test, which in contrast, all subjects could perform. To avoid floor effects as experienced on behavioral tests such as the OLSA for poor performing subjects, the logatome discrimination test provides a valuable clinical evaluation alternative.

For both the normal hearing control group and the good CI performers, the speech intelligibility results of the OLSA were clustered. The resulting SNR distribution for the normal hearing control group was rather narrow, while it was not possible to obtain results for the poor CI users. Thus, a categorization of the speech discrimination ability of the subjects was revealed by the OLSA.

The electrophysiological results support the speech intelligibility results revealed by the OLSA. No differences between the MMN amplitudes following vowel- and consonant-replacement paradigms were obtained. The similarity of the MMN amplitudes for normal hearing subjects and good CI performers, in contrast to that observed for the poor CI performers, justifies use of MMN component measures for screening for speech intelligibility and discrimination abilities. Beyond this categorization, the MMN so far appears not to be applicable to a direct measure of the discrimination abilities. Measurements of the MMN for CI users on a individual level are time consuming and difficult to confirm reliably[10]. Thus, this paper goes along with the conclusions of Kelly et al.[30], and Welge-Lüßen et al.[34] who describe limitations to the usefulness of the MMN to measure speech intelligibility. Nevertheless, the MMN appears as a possible complementary clinical tool to assess auditory sensitivity objectively[30,31,32]. Especially for patients who are not able to perform a behavioral test, the MMN should also be assessed in future tests.

As expected, the logatome discrimination test revealed a broad distribution of $d'$ for the CI users group and a narrow distribution for the normal hearing subject group. Thus, for the CI users, the logatome discrimination test allows a measure of speech discrimination abilities with a higher resolution than permitted by the OLSA or the MMN measure.

The logatome discrimination scores varied from chance level to almost 100% without evidence of a ceiling effect as observed and described for the HINT[40]. Thus, for the CI users, the logatome discrimination test seems to be ideally neither too easy nor too difficult. However, for normal hearing subjects, a clear ceiling effect was observed. This limits the applicability of the logatome discrimination test to the evaluation of limited speech perception abilities. In many cases, prelingually deafened CI users developed speech abilities through the use of hearing aids and by undergoing speech therapy. Consequently, they are able to perform commonly used clinical speech intelligibility tests[41]. Nevertheless, compared to speech identification, speech discrimination is a relatively simple task for prelingual subjects. Thus, the logatome discrimination test is applicable for testing auditory skills for many more individuals than the currently available speech intelligibility tests and is considered clinically appropriate for assessment of CI users at large. The majority of commonly available speech tests use syllables, words, or sentences as auditory stimuli[15,30,42]. However, much less is known about the applicability of logatomes to date. This paper demonstrates the use of the OLLO Corpus to design a powerful, psychoacoustic logatome discrimination test. As no differences between the discrimination of vowel- and consonant-replacement logatome paradigms (CVC) were found in our study, future tests will aim to assess discrimination ability using a VCV logatome paradigm also. Thereby, the spectrum of measurable contrasts should be extended by including different speakers[36]. Beside the speaking rate, speaker style, and speaker effort[35], prosody discrimination abilities[43] will also be assessed in future tests. From our point of view, the logatome discrimination test, with its wide number of test items, will provide a variable test corpus to measure basal speech discrimination skills of pre- and postlingual deafened CI users.

On the other hand, we also state that the MMN component measure is also reported as sensitive to logatome and syllable differences, and is therefore applicable to screen speech discrimination skills of uncooperative subjects for clinical purposes[9]. Furthermore, recent research suggests a high correlation between the MMN response and speech perception skills also beside the clinical focus[22]. Primarily due to the long recording times required to perform MMN, we propose the use of the MMN for clinical use in special cases only. While the logatome discrimination test is one example, for standard clinical purposes, we consider further development of quick and flexible psychoacoustical tests, based on the powerful OLLO Corpus.

## ACKNOWLEDGMENT

The research reported in this paper was funded by Cochlear Ltd. Europe. We also thank Josie Wyss and three anonymous reviewers for helpful comments on a previous version of the manuscript.
